# Dr. Henry Charles Procter

**Published:** 1910-04-09

**Authors:** 


### Obituary.

The death is announced at Neasden of Dr. Henry Charles Procter, formerly a medical practitioner in South Africa. During the Zulu war he was in charge of invalids from Natal to England, and had been district surgeon at Harrismith and Ladysmith.

